# Taking Impressions for Partial Sets of Teeth

**Published:** 1869-09

**Authors:** C. S. Chittenden


Taking Impressions for Partial Sets of Teeth.—By C. S. Chittenden.—In inserting partial sets of teeth, I always wish to do so with pressure plates. Of course I am sometimes compelled to resort to clasps, as it is, from the circumstances of an occasional case, impossible to make a pressure plate adhere with sufficient force to be satisfactory to myself or the patient. But, there need be but few such cases, if we could get a *perfect* impression of the parts. Usually, I am able to succeed with wax, but now and then a case is presented in which I find it necessary to adopt some other method of taking the impression, on account of the difficulty of removing the wax from the mouth, without changing the form of it. The following is the plan which I adopted some years ago, and which I have found to be the most successful, as well as the simplest method of any that I have tried. I first take an impression in wax and draw a cast from it. Then I take a piece of sheet lead and fit it to the cast as nearly as possible, by rubbing down with burnishers, something like a trial plate, leaving it a little higher on the edges than a plate which is to be worn, would bear to be. I use this lead pattern or trial plate, or whatever any one may choose to call it, for an impression cup, but as it would not be stiff enough to answer that purpose of itself I punch some small holes through the lead, and pour plaster of Paris over the whole of the lingual surface, letting it run through the small holes in the lead, so as to bind the plaster firmly to it, in the same way that mortar is bound to the lath on walls and ceilings. When the plaster has set I cut away all that will be in the way, and having prepared the plaster for the impression, I pour it into this made-up cup and putting it into the mouth I press it home in the usual way. I should say, however, that the plaster for the impression should be rather thin, so as to flow into all the spaces between the teeth. I allow it to set thoroughly before attempting to remove it, as it is less likely to break in doing so. Being far less thick and clumsy than the ordinary impression cup, almost any one can bear it in the mouth for ten or twelve minutes without much inconvenience, thus enabling me to take my own time in the removing of it from the mouth. In doing so, I first cut away, with a sharp pointed knife *all* the plaster from about the teeth, that I think will be likely to break away, and then, with some small instrument, very gently pry it away from the teeth, until it can be removed from the mouth without difficulty. From this impression I draw a cast and make the plate in the usual way, but of course, the teeth must be arranged in the *mouth*, as this is only an impression of the palate and lingual surfaces of the teeth.

[*Canada Dental Journal.*

